# Sex-linked placental DNA methylation associated with early-onset preeclampsia

**DOI:** 10.21203/rs.3.rs-10541872/v1

**Published:** 2026-08-13

**Authors:** Hannah J Illing, Icíar Fernández-Boyano, Amy M Inkster, Linda M Ernst, Gregory E Miller, Ann E Borders, Wendy P Robinson

**Affiliations:** BC Children’s Hospital Research Institute; BC Children’s Hospital Research Institute; BC Children’s Hospital Research Institute; Endeavor Health; Northwestern University; Endeavor Health; BC Children’s Hospital Research Institute

**Keywords:** Preeclampsia, placenta, DNA methylation, sex chromosomes, sex

## Abstract

Impaired placental development and function is a necessary feature of early-onset preeclampsia (EOPE), a severe pregnancy complication. We do not fully understand the molecular changes underlying EOPE; however, we know that DNA methylation, a chemical mark on DNA, can be measured to identify changes associated with EOPE. Typically, human cells have 22 pairs of autosomal chromosomes and one pair of sex chromosomes; females have two X chromosomes (XX) and males one X and one Y chromosome (XY). This is the first study to investigate and identify an association between EOPE and X and Y chromosome DNA methylation in the placenta. We used DNA methylation data from 843 placentas (XX = 386, XY = 457). We identified differences in X and Y chromosome DNA methylation associated with EOPE-pathology. Most differences in DNA methylation were observed in both males and females. Some of the associations were in genes which may have relevance to the pathophysiology of EOPE in the placenta. Our study highlights that there are important findings on the X and Y chromosomes that have been missed by their routine exclusion in previous studies.

## Plain English Summary

Impaired placental development and function is a necessary feature of early-onset preeclampsia (EOPE), a severe pregnancy complication. We do not fully understand the molecular changes underlying EOPE; however, we know that DNA methylation, a chemical mark on DNA, can be measured to identify changes associated with EOPE. Typically, human cells have 22 pairs of autosomal chromosomes and one pair of sex chromosomes; females have two X chromosomes (XX) and males one X and one Y chromosome (XY). This is the first study to investigate and identify an association between EOPE and X and Y chromosome DNA methylation in the placenta. We used DNA methylation data from 843 placentas (XX=386, XY=457). We identified differences in X and Y chromosome DNA methylation associated with EOPE-pathology. Most differences in DNA methylation were observed in both males and females. Some of the associations were in genes which may have relevance to the pathophysiology of EOPE in the placenta. Our study highlights that there are important findings on the X and Y chromosomes that have been missed by their routine exclusion in previous studies.

## Background

Preeclampsia (PE) is a leading cause of maternal and fetal morbidity and mortality with an estimated global prevalence of approximately 4% of pregnancies (1). PE is a pregnancy complication that presents with hypertension in addition to proteinuria, uteroplacental dysfunction, or other maternal-end organ dysfunction (2, 3). The pathogenesis of PE is incompletely understood, but impaired placentation (impaired spiral artery remodelling, abnormal trophoblast development, placental vascular dysfunction) is thought to be a key driver of the pathology (4, 5). This can lead to oxidative damage and the release of pro-inflammatory and anti-angiogenic factors into maternal blood, thus contributing to the development of maternal hypertension and multisystemic disease in the later trimesters of pregnancy (4, 6). Placentas from pregnancies complicated by PE have widespread differences in autosomal placental gene expression and DNA methylation (DNAme) patterns (7–13). PE associated placentas tend to have globally lower DNAme relative to normotensive controls, and there are more DNAme changes observed for early-onset (EOPE, diagnosed < 34 weeks) than late-onset (LOPE) cases (7, 13, 14) in comparison with gestational-age matched normotensive controls. No study to date has included the sex chromosome complement of the placenta in their analyses, despite there being a slight female-fetus bias in the incidence of EOPE (15).

Placental sex, defined by XX or XY sex chromosome complement, can influence development (15, 16), and exclusion of the sex chromosomes from DNAme studies means that important DNAme associations and sex differences could be missed. This exclusion is not specific to PE studies, and epigenome-wide association studies (EWAS) commonly exclude the X and Y chromosomes (17). This is attributable to differences in chromosome copy number, as well as X-chromosome inactivation (XCI), both of which can complicate analysis of the sex chromosomes and require a sex-stratified approach. There may also be an inherent bias in the research community that there are not interesting associations with these chromosomes. Nonetheless, the X chromosome (~ 154 Mb, ~ 5% of genome) contains more than 1,400 protein coding genes, including many genes related to immune-function (e.g., *IKBKG, WAS, FOXP3*) and microRNAs, which could be involved in PE (18). The Y chromosome (~ 62 Mb, ~ 1% of genome) is smaller and has about 700 genes, only 106 of which are protein-coding while the rest are pseudogenes (19–21). However, many Y-linked genes are expressed in the placenta including minor histocompatibility genes (*UTY, DDX3Y, RPS4Y1, KDM5D*, and *USP9Y*); of these, *UTY* and *KDM5D* are also implicated in histone modifications (22).

The exclusion of sex chromosomes from previous placental EWAS of EOPE means that important associations are potentially being missed and that sex differences in EOPE-linked DNAme have not been fully investigated. The first aim of this study was to identify differences in sex chromosome DNAme between placentas from pregnancies with a clinical diagnosis of EOPE (cases) and placentas from non-preeclamptic preterm births (controls). We proceeded to then identify sex chromosome DNAme associated with *eoPRED* score in a larger cohort of placentas. The eoPRED score is a DNAme-based continuous score, that was developed and validated as a measure of EOPE-related vascular placental pathology (23, 24). eoPRED uses DNAme at 45 CpGs identified through a machine-learning approach to score placental samples from zero to one, with higher scores corresponding to a higher probability of the placenta coming from a pregnancy with EOPE. Identifying DNAme associated with eoPRED score allows us to include more placentas in the analysis, as we are not limited to cases with clinical diagnoses, and enables us to consider placentas with pathological features that might not meet the clinical criteria for EOPE. This study is the first to identify sex chromosome DNAme associated with PE and expands our understanding of sex influences on placental epigenetic variation.

## Methods

### Datasets

To investigate sex chromosome DNAme associated with EOPE, we compiled DNAme array data from several publicly available datasets (**Figure S1**). We searched the Gene Expression Omnibus (GEO) with the keywords: “placenta” and “preeclampsia” with the filters for organism as “Homo sapiens” and study type as “Methylation profiling by array” or “Methylation profiling by genome tiling array”. There were 20 datasets in the search results that we reviewed further for our inclusion criteria: (i) human chorionic villi samples, (ii) profiled with the Infinium HumanMethylation450 BeadChip (450K) array (to minimize between-platform differences), with (iii) the inclusion of some cases of PE. Thirteen data sets were excluded due to wrong tissue type (n = 5), having no cases with PE (n = 3), duplicate samples contained in other data sets (n = 3), or different array platform (n = 2) (**Figure S1**). We combined the remaining 7 datasets into a single larger dataset (n = 379 samples) comprising the final ‘*450K Dataset’* (GSE100197 (n = 102)(7), GSE103253 (n = 82)(9), GSE98224 (n = 48)(7), GSE57767 (n = 45)(25), GSE125605 (n = 42) (10), GSE73375 (n = 36)(26), and GSE75196 (n = 24)(27)). We acknowledge that autosomal DNAme data from all seven of these datasets had previously been used to develop the eoPRED tool (23). However, sex chromosome data were not included in the training or testing of eoPRED, and cases and controls used in its development were balanced by sex.

We also utilized the Stress Pregnancy and Health Study (SPAH) dataset which was a single multi-ethnic cohort of 493 placental samples recruited in Chicago, Illinois, USA with Illumina Infinium MethylationEPIC BeadChip v1.0 (EPICv1.0) array data available also on GEO as GSE307289 (24, 28, 29). A detailed description of the SPAH data, including clinical characteristics, placental pathology, and DNAme data processing is provided in Beraldo *et al*. (28) and Illing *et al*. (24). Due to the comparatively few cases of EOPE, we used this dataset to investigate eoPRED score associations with the sex chromosomes. Two SPAH samples were excluded from analysis as the records did not indicate whether there was a diagnosis of PE. All other cases in SPAH had information about the presence/absence of a PE diagnosis.

### Preeclampsia clinical diagnosis

We retained the PE or non-PE labels that were assigned in the original studies for all samples, acknowledging that there were slight differences in clinical diagnostic criteria for PE between the original studies. All previous studies that differentiated EOPE and LOPE cases did so based on whether the diagnosis of PE was made less than 34 weeks gestation. In datasets where gestational age at diagnosis was unknown, we defined EOPE as PE cases with a gestational age at delivery of < 34 weeks, ensuring that only cases of EOPE would be included. The comparison group, non-preeclamptic preterm birth (nPTB) was defined by the absence of PE and gestational age at delivery of < 37 weeks.

### DNAme processing and quality control

All processing steps, quality control, and analyses were performed in R version 4.3.1 using the *minfi* and *ewastools* packages(30, 31). Samples were removed if they failed any of the following quality checks: duplicate samples (n = 8), total fluorescence intensity below two standard deviations of the dataset mean (n = 5), or low mean inter-sample correlation of DNAme beta (β) values (Pearson’s R < 0.95) (n = 2).

We screened for chromosomal imbalance (aneuploidy) in all samples with the *conumee2.0* package (32). For the conumee2.0 analysis, probes were grouped into 50kb bins of at least 15 probes. We normalized the fluorescence intensity in each sample against 10 random XY samples. We used a second set of 10 XY samples to cross-check the initial 10 reference XY samples. No samples were detected with inferred aneuploidy in the SPAH Dataset, while there were two samples with inferred chromosome abnormalities detected in the 450K Dataset: GSM2589548 (GSE98224) possible chromosome 16 duplication, and GSM2674422 (GSE100197, 46,XX,der(X) t(X;7)(p22.2;q21.1)/46,XX) which was previously reported (33). These samples were excluded from downstream analysis.

Both the 450K and SPAH array data were normalized with the *adjusted dasen* normalization algorithm, which is an improved normalization process for the sex chromosomes as it separately normalizes the X chromosome probes in XX and XY groups (34). The following probes were removed before analysis: autosomal probes, polymorphic and cross-hybridizing probes (35), probes with known manufacturing issues (36), sex chromosome probes that overlapped the X-transposed region or repetitive elements (17), and probes with a detection p-value > 0.01 in ≥ 5% of samples in a dataset. Y chromosome probes were only evaluated for failing detection p-value in XY samples (17). The final number of probes in each dataset are listed in Table 1.

### DNAme estimated variables

The *PlaNET* R package (37) was used to estimate cell composition, gestational age, and genetic ancestry. Cell composition was estimated as the proportions of the six major placental cell types: syncytiotrophoblast, cytotrophoblast, endothelial, stromal, Hofbauer, and nucleated red blood cells (nRBC), using the robust partial correlations method (38). PlaNET was also used to estimate gestational age (in weeks) for samples without a reported gestational age at delivery (450K Dataset n = 126) (39). For gestational age estimation we used the Robust Placental Clock, which was trained on > 1000 placental samples including some with PE, thus being the best suited version of the clock for these datasets. PlaNET also estimates compositional probabilities of European, East Asian, and African genetic ancestry, and the sum of all three probabilities equals to one, as other ancestries were not included in this model (40). In the linear models, correction for placental genetic ancestry was done by including two of the three continuous ancestry variables as additive covariates, rather than adjusting for reported maternal ethnicity, as genetic ancestry coordinates is more directly related to variation in placental DNAme.

PlaNET was also used to calculate eoPRED score, estimating a value between zero and one for each sample, which corresponds to the likelihood of coming from a pregnancy with EOPE (23). Previously, we defined a cutoff of 0.55 as having good discriminatory power to separate EOPE from nPTB cases. However, as we previously demonstrated the continuous eoPRED score also reflects the severity of maternal vascular malperfusion pathology which can be associated with low birthweight and preterm birth in the absence of PE (41). As PE diagnostic criteria varied between included studies, and there were some placentas with pathological features but without clinical diagnoses of EOPE we conducted an analysis evaluating sex chromosome DNAme associated with continuous eoPRED score in all samples.

### Placental sex

Placental sex was defined by an XX or XY sex chromosome complement, which was determined by i) the normalized fluorescence intensity of X and Y chromosome probes (17, 42), and ii) the average DNAme of the *XIST* promoter (43). XY samples had high (> 0.80) DNAme at these five probes in *XIST*: cg03554089, cg05533223, cg11717280, cg12653510, cg20698282, cg03862022, and cg23828566. Samples were removed from analysis if the DNAme-determined sex did not match reported fetal sex (n = 3, 450K Dataset) or if they had *XIST* probe DNAme intermediate to XX and XY group clusters which can be due to potential contamination with maternal tissue (n = 7, 450K Dataset) (**Table S1**).

### Principal Component Analysis

Sex-stratified principal component analysis (PCA) was performed separately for the X and Y chromosome β-values with the *irlba* packages (44). The *lmmatrix* function in the *plomics* package was used to identify covariance between the first four principal components (PCs) and metadata variables (45). We focused on the first four PCs, as higher PCs accounted for minor (< 5%) proportions of variance in β-values.

### Linear Modelling

To identify PE-associated DNAme we tested for associations between sex chromosome DNAme and EOPE clinical diagnosis (*Model 1*) or eoPRED score (*Model 2*) within each dataset and in sex stratified groups using multivariate linear models (*limma* package) (46). Linear modelling was performed on Mvalues (more homoscedastic) but estimates were reported in β-value units (Δβ) as they are more biologically interpretable (47). Gestational age and continuous genetic ancestry measures were included as covariates in the primary models (*Model 1a, Model 2a*) as they are potential confounders and major contributors to DNAme variation (39, 40, 48). As cell composition changes may contribute to EOPE pathology we ran additional models (*Model 1b, Model 2b*) which included PlaNET cell estimates (syncytiotrophoblast, stromal, endothelial, and nRBC) as covariates in the models. The cytotrophoblast estimate was excluded from the cell covariates as it was highly colinear (R^2^ ≥0.80) with the syncytiotrophoblast estimate, while the Hofbauer estimate was excluded as it made up < 1% of most samples. In all linear modelling with the 450K Dataset GEO dataset of origin was also included as a covariate as it was strongly associated with PC1.

We reported the total number of probes with false-discovery rate (FDR) q-value < 0.05 (FDR < 0.05) in all models. Based on a previous study that identified differences in DNAme between EOPE and nPTB groups we focused on probes with FDR < 0.05 and |Δβ|>0.1, which corresponded to at least a 10% difference in mean DNAme between EOPE and nPTB groups (7). In the eoPRED score models, we focused on probes with FDR < 0.05 and |Δβ|>0.25, which corresponded to at least a 2.5% difference in DNAme for a 0.1 change in eoPRED score. This was decided based on EOPE and nPTB groups in this study having a ~ 0.3 mean difference in eoPRED score.

### Identifying associations that are sex-specific

In sex-stratified analyses, observing significant effects in only one sex does not necessarily indicate presence of sex-specific effects, as this pattern is often due to chance sampling effects (49, 50). We thus used the strategy outlined in **Figure S2** to distinguish *sex-specific* X chromosome associations (i.e. an effect in one sex, but no effect in the other sex), *sex-differential* associations (i.e. a significant effect in both sexes but with different effect sizes), and *sex-opposite* associations (a significant effect in both sexes but with opposite effect directions) (Table 1). Steps to classify the type of association included determining if: i) the 95% confidence interval overlap of Δβ-values overlap between XX and XY groups; ii) regression coefficients are nominally significant (p < 0.05) in both groups; iii) regression coefficients have the same direction of effect in both groups; iv) the interaction term between EOPE/eoPRED and sex (EOPE:Sex or eoPRED:Sex) is significant. A *sex-specific* effect can be concluded if there was a significant association in only one sex, the confidence intervals do not overlap, and the interaction term was significant (51, 52). If an association was significant in both sexes, but the interaction between the EOPE/eoPRED term and was also significant, it would be classified as either *sex-differential* or *sex-opposite*, depending on the direction of association. If the regression coefficient was significant (p < 0.05) in only one sex, but the interaction test was not significant and the confidence intervals overlapped, we considered this as “*ambiguous*”. This could be a false positive in one sex or false negative in the other.

### Comparing between linear models

We compared regression coefficients (Δβ-values) between the EOPE vs. nPTB and eoPRED score models in the 450K Dataset using Pearson correlation tests. To identify a list of high-confidence, large-effect size associations with eoPRED score we compared the regression coefficients for the eoPRED score models between the 450K and SPAH Datasets at the 8,350 X chromosome probes and 198 Y chromosome probes that overlapped between the two array platforms. A ‘replicated DMC’ was defined as a probe with FDR < 0.05 in at least one dataset, nominal p < 0.05 in the other dataset, and |Δβ|>0.25 in at least one dataset.

### Functional Analysis

Using the *Bizet et al*. annotation, probes were mapped to genes and regulatory regions (Promoter, Enhancer, Dual (promoter and enhancer), Gene Body) (53). Functional analysis was performed with the KnowYourCG package (54) on all probes from Model 2a with nominal p-value < 0.05 in both datasets, FDR < 0.05 in at least one dataset, Δβ-values in the same direction, and |Δβ|>0.1 in at least one dataset (n = 580 X, n = 13 Y). Using the *testEnrichment* function we tested for enrichment in the following databases: chromHMM consensus from Roadmap, Transcription Factor Binding Site (TFBS) and consensus histone modifications from Cistrome/ENCODE, and CpG islands (EPIC hg38 manifest). Terms satisfying FDR < 0.01 were considered significantly enriched.

## Results

### Dataset characteristics

We used two datasets to identify DNAme variation on the X and Y chromosomes associated with EOPE pathology (Table 2). The “450K Dataset” consisted of 352 (48% XX) placentas profiled with the Illumina Infinium 450K array, and the SPAH Dataset consisted of 491 (44% XX) profiled with the EPIC v1.0 array. The 450K Dataset included 80 EOPE and 54 nPTB placentas, as many of the composite studies focused on PE and enriched for complicated pregnancies, while the SPAH Dataset contained 7 EOPE and 40 nPTB cases. The proportion of EOPE and nPTB cases did not differ by sex in either dataset, though nPTB cases did have slightly higher mean gestational ages than the EOPE cases in both datasets. Accordingly, in our linear models we included gestational age as a covariate.

In addition to identifying differences in DNAme between the EOPE and nPTB groups defined based on clinical presentation, we also identified DNAme associated with eoPRED score. This allowed us to use all samples in both the 450K and SPAH Datasets and has utility as placentas can show EOPE-related pathology without a clinical diagnosis of EOPE. The 450K Dataset had a total of 85 (24.1%) samples with eoPRED scores > 0.55, compared to only 33 (6.7%) in the SPAH Dataset (Table 2). However, using eoPRED score as a continuous rather than categorical variable may reflect the severity of placental vascular pathology (24) and thus, we used the score as a continuous measure of EOPE-associated placental dysfunction in subsequent analyses. The range of eoPRED scores was similar in both datasets and median eoPRED score did not differ by sex (Table 2, **Figure S3a, b**). We confirmed that placentas from pregnancies with EOPE had higher eoPRED scores than nPTB cases in both 450K and SPAH Datasets, but we note there were some late-onset PE cases and non-PE cases with scores > 0.55 (**Figure S3c**).

### Sex chromosome DNAme variation

PCA was performed to characterize variables associated with sex chromosome DNAme variation, and to evaluate the influence of technical and biological variables (**Figure S4**). In the 450K Dataset PCs 1 and 2 were strongly associated with GEO dataset and cell-type estimates. Genetic ancestry probabilities and gestational age were also associated with the first four PCs but not as strongly(R^2^ < 0.15). In the SPAH Dataset PCs 1 and 2 were strongly associated with the cell-type estimates. Based on these analyses, and a priori knowledge of variables associated with placental DNAme variation, downstream linear models were adjusted for GEO dataset, gestational age, and genetic ancestry. As cell composition could influence EOPE pathology, we elected to run models both with and without adjustment for cell composition.

### Differences in sex chromosome DNAme between EOPE and nPTB

First, we looked for EOPE-associated DNAme on the X and Y chromosomes in the 450K Dataset using the clinical diagnoses of EOPE (n = 40 XX; n = 40 XY) and nPTB (n = 23 XX; n = 31 XY). In Model 1a at FDR < 0.05 there were notably more probes differentially methylated on the X chromosome in the XY (n = 184) as compared to XX (n = 20) group (Table 3, Fig. 1a). There were 6 probes that also reached a biological threshold of |Δβ|>0.1 (Fig. 1a,b) all of which were located in cis-regulatory regions (promoters or enhancers) (**Table S2**). Five of these 6 probes did not show clear sex-specific effects (were considered ambiguous) as the confidence intervals were overlapping and/or the test for interaction was non-significant. However, cg19022384, which maps to an intergenic long non-coding RNA *LINC02931*, showed a significant interaction by sex, with a ‘sex-opposite’ effect: XY samples had lower DNAme in EOPE Δβ=−0.114, while XX samples exhibited higher DNAme in EOPE Δβ = 0.112 (Fig. 1b, **Table S2**).

Adjustment for cell composition (Model 1b) reduced the number of probes associated with EOPE in XX and XY samples (Table 3). At FDR < 0.05 there remained only 34 differentially methylated X chromosome probes in XY samples and no differentially methylated probes in XX samples. However, two probes (cg19022384, cg24268343) from Model 1a remained significant at |Δβ|>0.1 in Model 1b. No probes were differentially methylated between EOPE and nPTB groups on the Y chromosome before or after adjustment for cell composition.

### eoPRED score associations with sex chromosome DNAme in the 450K Dataset

We also evaluated DNAme patterns associated with continuous eoPRED score, as it allowed us to increase our power to detect effects by considering the placental phenotype on a continuum and thus using all samples in both datasets. Again, we saw many more associations on the X chromosome in XY as compared to XX samples (Table 3; Fig. 2a). In the 450K Dataset in Model 2a at FDR < 0.05 there were 343 and 1636 probes differentially methylated on the X chromosome in XX and XY samples, respectively. Of these, 10 X chromosome probes met a stricter effect size threshold of |Δβ|>0.25, two in XX and eight in XY samples. Some examples of these DMCs are shown in Fig. 2b. In addition, at FDR < 0.05 28 probes were associated with eoPRED score on the Y chromosome. Two of these 28 probes, both in the pseudogene *TXLNGY*, met the stricter |Δβ|>0.25 cutoff.

To identify the specific patterns of sex association, we focused on the 10 X chromosome probes that met FDR < 0.05 and |Δβ|>0.25. Of these 10 probes, six had an association in the same direction in both sexes and did not have a significant eoPRED*Sex interaction term, indicating a similar effect in both sexes (**Table S3**). The remaining four probes all were significant for interaction with sex. Three of these were thus considered to be sex-specific, with cg07824128 only significant in XX and cg22844265 and cg04238575 only significant in XY samples. cg16520030 was considered sex-differential in that it was significant in both XX and XY placentas but there was a greater effect in XY placentas. We observed similar results, albeit attenuated, in this dataset after adjustment for cell composition (Model 2b). In Model 2b there were still 10 probes associated with eoPRED score at FDR < 0.05 and |Δβ|>0.25, with 9/10 of those being the same probes identified in Model 2a.

### eoPRED score associations with sex chromosome DNAme in the SPAH Dataset

Due to differences in array platform and to focus on a single cohort uniformly sampled, we separately analyzed the SPAH Dataset, and similarly to the 450K Dataset there were more probes associated with eoPRED score on the X chromosome in the XY than XX group (Table 3). In Model 2a at FDR < 0.05 we observed 779 X chromosome probes associated with eoPRED score in XX samples, and 3,358 in XY (Fig. 3a). At FDR < 0.05 and |Δβ|>0.25, 47 unique probes remained significant including: four in XX samples and 44 in XY samples, with cg19321437 reaching significance in both sexes (**Table S4**).

Four of the 47 X chromosome probes associated with eoPRED score in the SPAH dataset at strict FDR < 0.05 and |Δβ|>0.25 cutoffs had XY-specific associations, none had XX-specific associations. Twenty four of the 47 X chromosome probes exhibited sex-differential associations, with all effect sizes larger in XY than XX samples, suggesting that the effects were also present in XX samples but were of smaller magnitude and harder to detect. Two of the 47 X chromosome probes were classified as ambiguous. The remaining 17/47 X chromosome probes did not show a significant interaction term and confidence intervals overlapped between sexes, suggesting there was a similart association in both sexes, despite only reaching FDR significance in one sex. Examples of each category of these findings are shown in Fig. 3b.

### Comparing models using clinical diagnosis of EOPE vs. eoPRED score

We previously demonstrated that eoPRED score is strongly associated with the clinical diagnosis of EOPE (23, 24). To further extend that conclusion to this analysis, we compared the regression coefficients of all probes from the EOPE versus nPTB models to coefficients from the same probes obtained in the eoPRED score models. We expected the model results to be similar despite differences in variables and sample sizes. The Δβ-values for Models 1a (EOPE) and 2a (eoPRED) in the 450K Dataset were highly linearly correlated at R = 0.76 on the X chromosome in XX samples (**Figure S5a**) and R = 0.58 on the X chromosome in XY samples (**Figure S5b**). Correlations were of slightly lower magnitude between cell-adjusted Models 1b (EOPE) and 2b (eoPRED) (R = 0.42 in both XX and XY samples), suggesting that part of the shared signal between eoPRED score and EOPE clinical diagnosis may be attributable to differences in cell composition. Considering the Y chromosome, regression coefficients between Models 1a (EOPE) and 2a (eoPRED) were moderately correlated (R = 0.29) (**Figure S5c**), with a similar attenuation of the correlation strength between cell-adjusted Models 1b and 2b (R = 0.19). In general, regression coefficients from the eoPRED models (2a, 2b) tended to be larger in absolute magnitude than regression coefficients from the EOPE versus nPTB models. Differences between EOPE and eoPRED models are likely due to a combination of factors, including differences in sample size (statistical power), use of a categorical versus continuous predictor, and perhaps different underlying biological changes captured by eoPRED score that differ from the features reflected in a clinical diagnosis of EOPE.

### Replicated eoPRED score DMCs

As the first large-scale study of eoPRED score-associated DNAme patterns, we sought to identify a list of high-confidence and large effect size ‘replicated eoPRED DMCs’ shared between the 450K and SPAH datasets. Across the two datasets there were a total of 15 unique ‘replicated eoPRED DMCs’ (13 X chr, 2 Y chr), which represented 46.9% of the initial DMCs identified in both datasets (Table 4, **Figure S6**). These CpGs were associated with 12 different genes including several with known relevance in placenta, e.g. *ACE2* (angiotensin-converting enzyme 2)(55), *MBNL3* (Muscleblind-like protein 3)(56) and *MIR503HG* (57, 58). Thirteen of the DMCs showed a consistent relationship in XX and XY groups in both datasets. The replicated DMP cg22683858 in *CHIC1* was unusual because although it was detected in both 450K and SPAH datasets it was only associated with eoPRED score in XX samples, though the Δβ 95% confidence intervals overlapped between the sexes in both datasets and there was no significant interaction between eoPRED and sex. Interestingly, this gene lies very close to the X-inactivation centre on the X chromosome although has no known function in placenta. This probe thus did not meet the criteria to be called a sex-specific or sex-differential association but did appear to be consistently associated with eoPRED score in XX samples in both datasets. Follow-up in an independent cohort of placentas may be necessary to clarify the sex-specificity of this association. cg22844265 was another replicated eoPRED-associated X chromosome probe that was classified as XY-specific in the 450K Dataset, while in the SPAH Dataset it was classified as sex-differential (XY > XX effect). As the interaction term was significant for this probe in both datasets, we concluded that sex modifies the effect of the association between eoPRED and DNAme at this locus but could not confidently call it a sex-specific association.

Considering the 15 probes associated with eoPRED score in both the 450K and SPAH datasets, we subsequently performed a functional analysis to assess for any trends in gene function, transcription factor binding sites (TFBS), or probe position relative to CpG islands (CGIs). As no associations reached significance to characterize the 15 replicated DMCs, we loosened the inclusion criteria to look at all replicated DMCs with a |Δβ|>0.1 in at least one dataset (580 X chr probes, n = 13 Y chr probes) to characterize cross-dataset patterns of eoPRED-associated DNAme. The 580 cross-dataset eoPRED-associated X chromosome probes were enriched for the following terms: repressive polycomb state (13_ReprPC), CpG island, N shore of CpG islands, and the TFBS of *ATF7IP* (Activating transcription factor 7-interacting protein 1) (**Figure S7, Table S5**). ATF7IP is important for SETDB1 deposition of H3K9me3 and the maintenance of XCI(59). Collectively, these terms indicate enrichment for probes located in repressed X chromosome CpG islands. There were no terms enriched for the Y chromosome probes, but there were few probes included, and/or fewer protein coding Y chromosome genes.

## Discussion

Autosomal placental DNAme patterns associated with PE have been well studied (7–13). To our knowledge, no study has yet included the X and Y chromosomes in EOPE-associated DNAme patterns. While the X and Y chromosome data are collected at the same time as autosomal data on the DNAme microarrays, the sex chromosomes are often excluded from analyses. This study sought to address this gap by identifying EOPE-associations with sex chromosome DNAme.

We used two different strategies to identify EOPE-associated DNAme, first modeling EOPE versus nPTB, and second modeling continuous DNAme-derived eoPRED scores, with higher scores reflecting higher likelihood of EOPE. Some advantages of using eoPRED score included being able to include more samples in the analysis, thus increasing statistical power. Second, as EOPE diagnostic criteria varied between studies, we hypothesize that eoPRED score may be more optimal for comparing across diverse datasets. Finally, since eoPRED score is a continuous variable, it may more accurately represent the clinical spectrum of EOPE-associated placental pathology, as opposed to the binary clinical classification of EOPE versus nPTB. Overall, effect sizes were highly correlated between models using the clinical diagnosis of EOPE and those using eoPRED score, reinforcing the utility of eoPRED score in place of clinical diagnoses for identifying PE-associated DNAme variation when the clinical diagnosis information is unavailable or researchers are interested in capturing the underlying pathology as a continuous variable.

In addition to identifying sex chromosome DNAme associated with EOPE, we also assessed whether sex moderated these associations. Using our decision tree (Fig. 1) most of the X chromosome probe associations observed were not sex-specific, rather the associations were either seen in both sexes or were sex-differential. Where there was a sex-differential association, the effect sizes were typically larger in XY than XX placentas. Furthermore, there were typically more X chromosome probes meeting significance in association with eoPRED score in XY versus XX samples – yet this did not correspond with more XY-specific DNAme differences when our decision tree criteria were examined, as many of these probes showed eoPRED effects in the same direction in both sexes, but only reached significance in the XY samples.

DNAme associated with X-chromosome inactivation (XCI) likely masks or reduces the detectable DNAme effect sizes in XX samples. Since the Illumina microarrays do not provide allele-specific DNAme information, the β-values of the X chromosome in XX samples are the average of the active X (Xa) and inactive X (Xi). While the Xa is thought to have a similar DNAme profile to the single X chromosome in XY samples, the Xi has higher mean levels of DNAme, concentrated at promoters and CpG islands (60). Thus, there is an averaging of X-linked DNAme in XX samples between high and low-methylated X chromosomes, meaning that the magnitude of associations in XX samples are expected to be roughly half that observed in XY samples if the region is subject to XCI-associated DNAme. This ‘XCI masking’ has been observed in genetic association studies, where there was twice as much X-linked genetic variance associated with complex-traits in males than females (61, 62). This phenomenon underscores the importance of utilizing appropriate statistical methods (interaction and stratified tests) to report sex-differential and sex-specific effects (63), as effects detected in only males may also be present in females but with smaller effect sizes, or may miss statistical significance. Moreover, at the gene expression level the effect is likely similar between sexes since generally only one X is expressing these genes in either sex. Furthermore, X chromosome DNAme in the placenta is unique compared to fetal tissues (64). Compared to somatic tissue, CpG island promoters on the Xi have lower DNAme in the placenta, although there does not seem to be more escape from XCI (64, 65). Further exploration into factors influencing sex chromosome DNAme variation and the differences between XX and XY placentas is needed.

While EOPE affects more pregnancies carrying XX than XY fetuses (15) we did not identify major sex differences in EOPE-associated placental DNAme on the X chromosome. Sex differences in DNAme have been identified on autosomes (48, 66, 67) and could also play a role EOPE, although we previously found little evidence that EOPE-associated autosomal DNAme differed by fetal sex (68). Sex differences in PE are likely complex and could arise also from sex differences in gene expression or sex hormone levels which may not be associated with changes in DNAme (e.g. PMID 38600394). For instance, all seven genes in the *LHB-CGB* cluster on chromosome 19, are higher expressed in XX placentas (69). A subset of these genes encode subunits for human chorionic gonadotropin (hCG), which is present at higher levels in maternal blood when the fetus is female as early as 3 weeks GA(70) and the mRNA for its subunits but its unknown if there is any interaction between these sex differences and early placentation leading to EOPE. In addition, lower levels of estradiol, a sex hormone involved in placental vasculogenesis, have been observed in the blood of pregnant individuals with PE, but it is unknown whether feto-placental sex modifies this association (71, 72).

With respect to the biological meaning of X and Y eoPRED-associated DMPs, several are located in genes that may be relevant to PE pathophysiology. For example, cg08559914 is a sex-differential X chromosome probe (Δβ XY > XX) located within the enhancer region of *ACE2*, a gene which is expressed in the syncytiotrophoblast cell layer. ACE2 is a membrane protein that converts angiotensin II to angiotensin (1–7) which is involved in inhibiting inflammation, oxidative stress, and vasoconstriction (73). The level of soluble ACE2 in maternal blood has been reported to be higher at mid-gestation (~ 20 weeks) and lower at term in pregnancies complicated by PE(74, 75). In mice, *Ace2* knockouts had PE-associated phenotypes including maternal hypertension, placental hypoxia, and fetal growth restriction(76). Another sex-differential X chromosome probe (cg16520030; Δβ XY > XX), is located within intron 2 of *MBNL3* which is expressed in trophoblast cells and only minimally expressed in other tissues (56). Knockout of *Mbnl3* in mice embryos resulted in increased placental weight (56). Two replicated DMCs (cg20978230, cg07924363) are located within the placentally expressed long non-coding RNA *MIR503HG* (host gene to miR-503 and miR-424). Expression of *MIR503HG* was upregulated in placentas from PE cases, and increased expression of *MIR503HG* inhibited trophoblast cell proliferation (57, 58). Inversely, miR-424 expression was downregulated in PE and miR-424 upregulation improved trophoblast invasion and migration (77). Dysregulation of *ACE2, MBNL3*, and *MIR503HG* placental expression through altered DNAme could contribute to placental vascular dysfunction observed in PE.

Although the Y chromosome is rarely considered in EWAS studies, some Y-linked genes are highly expressed in the placenta. We observed two Y chromosome differentially methylated probes in the gene body in the pseudogene taxilin gamma Y-linked (*TXLNGY*). Although there is no known function of this pseudogene, it is ubiquitously expressed in most adult tissues in males, including placenta (78, 79) and may be expressed in placental endothelial cells (80).

Strengths of this study included our use of two large placental datasets to both identify and validate sex chromosome differences in DNAme. Despite the datasets being profiled with different array platforms and being recruited from different populations, there was a high correlation of regression coefficients between the datasets, indicating overall between-cohort consistency in the reported eoPRED associations. A limitation of this study is that reported gestational age was not available for all samples, for which the PlaNET epigenetic gestational ages were used instead. The median error of the PlaNET robust placental clock is one week, although, there is a slightly higher error rate for preterm samples (39, 81). Another limitation of this study is that although some probes were called sex-differential or sex-specific, the magnitude of the difference between sexes may not be very large or biologically meaningful (63). We also acknowledge that the 450K Dataset was comprised of samples used to train eoPRED, thus we could be overfitting the data when calculating eoPRED score for those samples. However, the use of the SPAH Dataset indicates the robustness of findings in an independent cohort.

### Perspectives and Significance

We provide an approach for categorizing DNAme associations involving the sex-chromosomes that can be applicable to studies involving other tissues and contexts. Although in sex stratified association studies one often observes statistical significance in one sex or the other, most such observations are not “sex-specific”. It is though common to see a large effect size in XY as compared to XX samples, due to masking of effects from DNAme associated with XCI; this may not hold at the gene expression level since there is only one active X in both sexes. There are many genes on the X chromosome that function in development and these should not be ignored when investigating the molecular basis of disease.

## Conclusions

Our study indicates that there are robust EOPE-associated differences in DNAme on the sex chromosomes in the human placenta. We observed more X chromosome DMCs in XY than XX samples, likely because of XCI masking the effect-size of associations in XX samples. Although many DMCs were potentially associated with pathology-related cell composition changes, some remained significant even after correction for cell composition. We detected similar differences in DNAme whether we used the clinical diagnosis of EOPE or placental pathology (eoPRED score). However using eoPRED score allowed us to increase the power in our analyses and detect more DMCs. As evidenced by the findings in this paper, the sex chromosomes should be included in future placental epigenomic analyses, as our work has identified potentially important sex-linked associations with EOPE.

## Supplementary Material

Supplementary Files

This is a list of supplementary files associated with this preprint. Click to download.

- AdditionalFile1SupplementaryFigures.pdf
- AdditionalFile2SupplementaryTables.xlsx

## Figures and Tables

**Figure 1:**
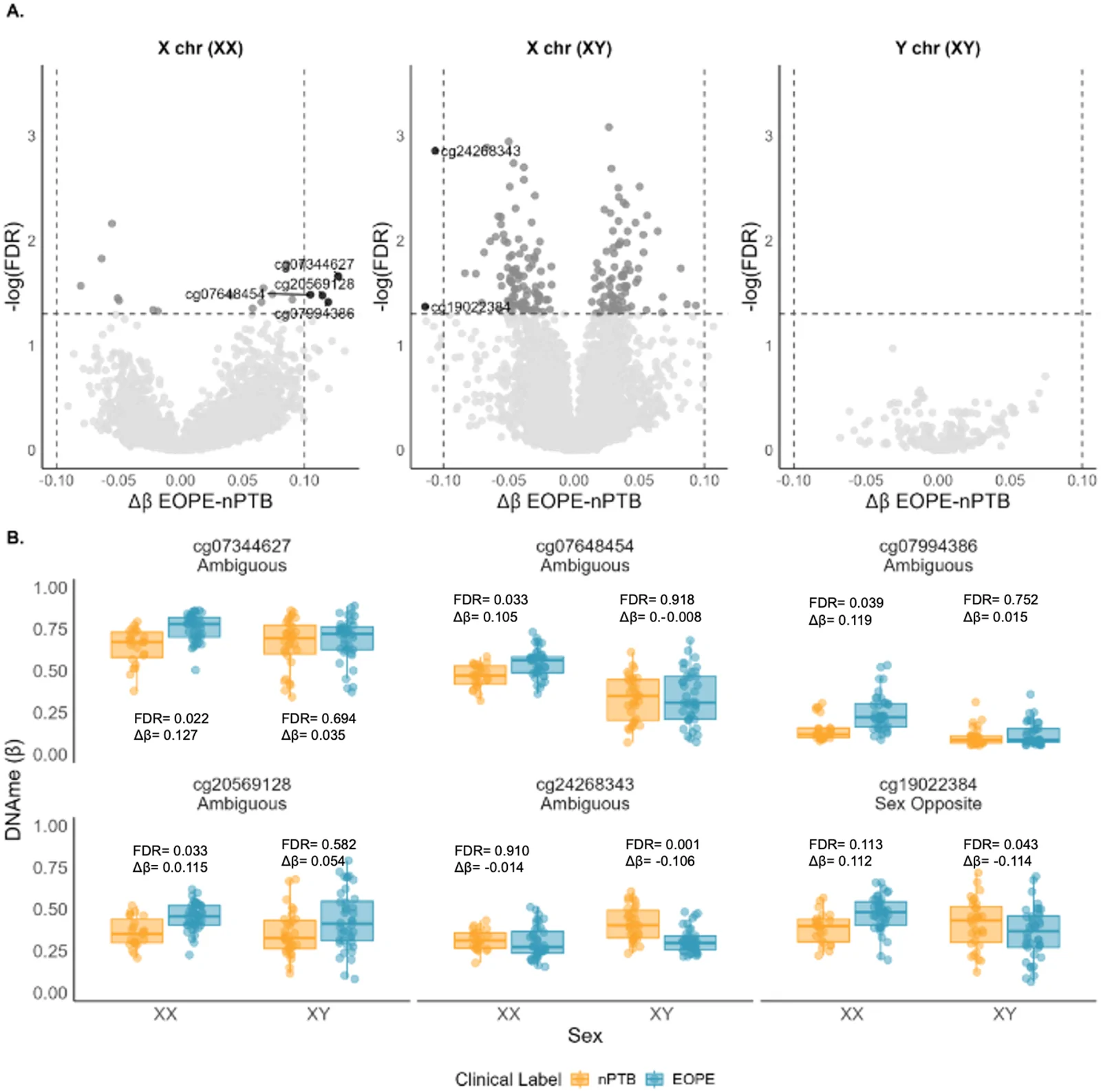
DNAme differences between EOPE and nPTB groups in the 450K Dataset **A** Volcano plots from DNAme~EOPE vs. nPTB without cell-types as covariates(*Model 1a*); The horizontal grey dotted line indicates an FDR cutoff of 0.05, while the horizontal grey dotted lines indicate the (| Δβ|>0.1) threshold. **B** Box plots for DNAme at the 6 X chromosome significant CpGs in at least one sex. FDR and Δβ-values come from the linear regressions.

**Figure 2:**
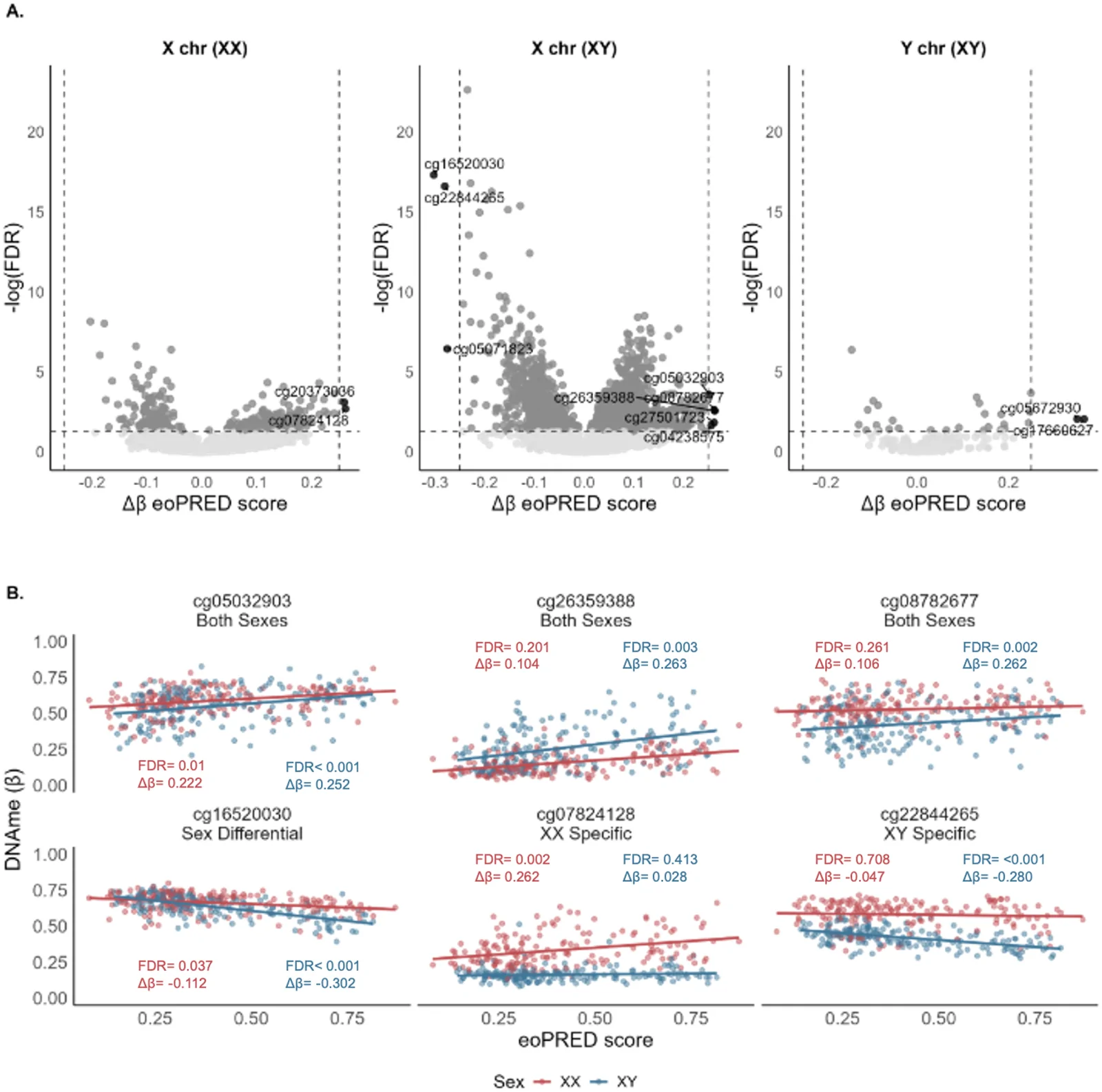
DNAme associated with eoPRED score in the 450K Dataset **A** Volcano plots for DNAme ~ eoPRED score without cell-types as covariates (*Model 2a*). **B** Scatter plots showing DNAme vs. eoPRED score for 6 X chromosome DMCs coloured by sex. The type of association (both sexes, sex differential, sex-specific) is indicated. FDR and Δβ-values come from the linear regressions.

**Figure 3:**
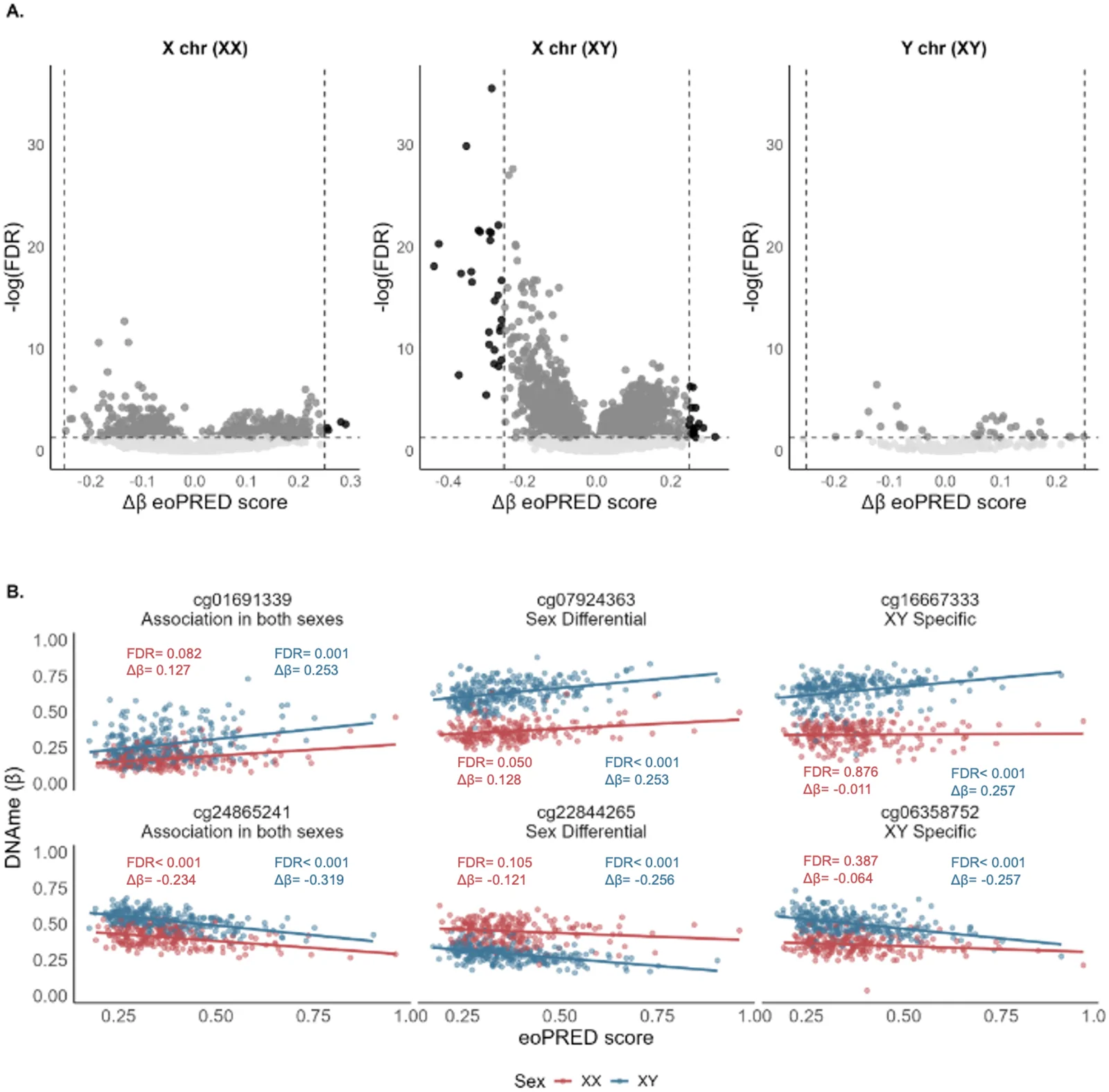
DNAme associated with eoPRED score in the SPAH Dataset **A** Volcano plots for DNAme ~ eoPRED score (*Model 2a*). **B** Scatter plots showing DNAme vs. eoPRED score for 6 X chromosome DMCs coloured by sex. The type of association (both sexes, sex differential, sex specific) is indicated. FDR and Δβ-values come from the linear regressions.

**Table 1:** Classifying sex-linked associations with FDR < 0.05 in at least one sex

Criteria	Sexspecific	Sexopposite	Sex-differential	Both Sexes	Ambiguous*
Δβ 95% CIs overlap	No	No	No	**Yes**	**Yes**
Regression nominal p < 0.05 in both sexes	No	**Yes**	**Yes**	**Yes**	No
One CI overlaps 0	**Yes**	No	No	No	**Yes**
CIs are in the same direction	--	No	**Yes**	**Yes**	--
eoPRED:Sex interaction q < 0.05	**Yes**	**Yes**	**Yes**	No	No

CI=confidence interval;

* p < 0.05 in only one sex, but values do not differ by sex, suggesting a false positive or negative.

**Table 2:** 450K and SPAH Dataset demographics

	450K Dataset		SPAH Dataset (EPICvl)	
GEO Accession IDs	GSE100197, GSE103253, GSE98224, GSE57767, GSE125605, GSE73375, GSE75196		GSE307289	
**N**	352		491	
**Placental Sex**	XX (n = 169)	XY (n = 183)	XX (n = 217)	XY (n = 274)
**Clinical Diagnosis (n (%))**				
Early-onset PE (EOPE)	40 (23.7)	40 (21.9)	3 (1.4)	4 (1.5)
Non-PE Preterm birth (nPTB)	23 (13.6)	31 (16.9)	14 (6.5)	26 (9.5)
**Gestational age (weeks)**				
All	36.1 (3.8)	35.9 (3.6)	38.7 (19)	38.5 (2.1)
EOPE	31.7 (3.3)	31.7 (3.3)	29.9 (5.2)	30.5 (2.2)
nPTB	32.8 (3.6)	33.5 (2.8)	34.9 (16)	34.8 (2.5)
**eoPRED median score** [IQR]	0.35 [0.26,0.55]	0.35 [0.27–0.54]	0.35 [0.29–0.41]	0.34 [0.28–0.43]
**eoPRED > 0.55** (n (%))	42 (24.9)	43 (23.5)	17 (7.8)	16 (5.8)
**Probes**				
X Chromosome	9,210		16,070	
Y Chromosome	226		289	

**Table 3:** Linear modelling results. The table lists the numbers of probes with FDR < 0.05 from the linear modelling for EOPE vs nPTB (Model 1) and eoPRED (Model 2). Model 1a and 2a adjust for cohort, gestational age and ancestry; while Model 1b and 2b additionally adjusts for cell composition.

450K Dataset: EOPE vs nPTB (categorical)			
Model	X chr (XX)	X chr (XY)	Y chr (XY)
	EOPE = 40 vs. nPTB = 23	EOPE = 40 vs. nPTB = 31	EOPE = 40 vs. nPTB = 31
1a	20	184	0
1b	0	34	0
450K Dataset: eoPRED score			
Model	X chr (XX)	X chr (XY)	Y chr (XY)
	n = 169	n = 183	n = 183
2a	343	1,636	28
2b	53	570	7
SPAH Dataset: eoPRED score			
Model	X chr (XX)	X chr (XY)	Y chr (XY)
	n = 217	n = 274	n = 274
2a	779	3,358	35
2b	94	872	2

**Table 4:** Replicated sex chromosome DMCs associated with eoPRED score

Probe	Sex-initially identified in	Position (hg38)	Gene Name	Regulatory Region	Call
**X chromosome**					
cg22683858	XX	73562961	*CHIC1*	Promoter	Ambiguous
cg19321437	XX & XY	84502527	*HDX*	Promoter	Both sexes
cg08559914	XY	15602116	*ACE2*	Enhancer	Sex-differential
cg10840597	XY	47918271	*ZNF81*	Gene body	Both sexes
cg02212907	XY	55162450	*VCF2*	Intergenic	Sex-differential
cg22844265	XY	69151399	*PJA1*	Enhancer	XY-specific (450K)/Sex-differential (SPAH)
cg05071823	XY	118494707	*DOCK11*	Enhancer	Both sexes
cg19227523	XY	84502527	*XPNPEP2*	Enhancer	Sex-differential
cg00422452	XY	129738084	*XPNPEP2*	Enhancer	Sex-differential
cg26359388	XY	131792574	*FIRRE*	Promoter	Both sexes
cg16520030	XY	132415021	*MBNL3*	Enhancer	Sex-differential
cg20978230	XY	134546338	*MIR503HG*	Dual	Both sexes
cg07924363	XY	134546781	*MIR503HG/MIR424*	Dual	Sex-differential
**Y chromosome**					
cg05672930	XY	19567668	*TXLNGY*	Gene body	
cg17660627	XY	19567778	*TXLNGY*	Gene body	

## Data Availability

All datasets analyzed during the current study are available in the Gene Expression Omnibus (GEO): GSE100197, GSE103253, GSE9822, GSE57767, GSE125605, GSE73375, GSE75196, and GSE307289. The R code to support this article is available at https://github.com/hanilling/Sex-chromosome-DNAme-associated-with-EOPE.

## Highlights

- This is the first study to identify EOPE associations on the sex chromosomes
- Most X chromosome associations were not sex-specific, but were present in both sexes
- X-chromosome inactivation may reduce the effect size of X chr DMCs in XX samples
- DMCs in X-linked genes *ACE2* and *MBLN3* may be relevant to the pathophysiology of EOPE
